# Predicting risk of metastases and recurrence in soft-tissue sarcomas via Radiomics and Formal Methods

**DOI:** 10.1093/jamiaopen/ooad025

**Published:** 2023-04-12

**Authors:** Roberto Casale, Giulia Varriano, Antonella Santone, Carmelo Messina, Chiara Casale, Salvatore Gitto, Luca Maria Sconfienza, Maria Antonietta Bali, Luca Brunese

**Affiliations:** Department of Radiology, Institut Jules Bordet—Université Libre de Bruxelles (ULB), Brussels, Belgium; Department of Medicine and Health Sciences Vincenzo Tiberio, University of Molise, Campobasso, Italy; Department of Medicine and Health Sciences Vincenzo Tiberio, University of Molise, Campobasso, Italy; IRCCS Galeazzi Orthopedic Institute, Milan, Italy; Department of Biomedical Sciences for Health, University of Milan, Milan, Italy; Allergology Service, Dermatology Unit, Azienda Ospedaliera Universitaria di Modena, Modena, Italy; IRCCS Galeazzi Orthopedic Institute, Milan, Italy; Department of Biomedical Sciences for Health, University of Milan, Milan, Italy; IRCCS Galeazzi Orthopedic Institute, Milan, Italy; Department of Biomedical Sciences for Health, University of Milan, Milan, Italy; Department of Radiology, Institut Jules Bordet—Université Libre de Bruxelles (ULB), Brussels, Belgium; Department of Medicine and Health Sciences Vincenzo Tiberio, University of Molise, Campobasso, Italy

**Keywords:** Formal Methods, magnetic resonance imaging, metastases, model checking, Radiomics, soft-tissue sarcoma

## Abstract

**Objective:**

Soft-tissue sarcomas (STSs) of the extremities are a group of malignancies arising from the mesenchymal cells that may develop distant metastases or local recurrence. In this article, we propose a novel methodology aimed to predict metastases and recurrence risk in patients with these malignancies by evaluating magnetic resonance radiomic features that will be formally verified through formal logic models.

**Materials and Methods:**

This is a retrospective study based on a public dataset evaluating MRI scans T2-weighted fat-saturated or short tau inversion recovery and patients having “metastases/local recurrence” (group B) or “no metastases/no local recurrence” (group A) as clinical outcomes. Once radiomic features are extracted, they are included in formal models, on which is automatically verified the logic property written by a radiologist and his computer scientists coworkers.

**Results:**

Evaluating the Formal Methods efficacy in predicting distant metastases/local recurrence in STSs (group A vs group B), our methodology showed a sensitivity and specificity of 0.81 and 0.67, respectively; this suggests that radiomics and formal verification may be useful in predicting future metastases or local recurrence development in soft tissue sarcoma.

**Discussion:**

Authors discussed about the literature to consider Formal Methods as a valid alternative to other Artificial Intelligence techniques.

**Conclusions:**

An innovative and noninvasive rigourous methodology can be significant in predicting local recurrence and metastases development in STSs. Future works can be the assessment on multicentric studies to extract objective disease information, enriching the connection between the radiomic quantitative analysis and the radiological clinical evidences.

## BACKGROUND AND SIGNIFICANCE

Soft-tissue sarcomas (STSs) are a group of malignancies which include a wide number of subtypes, all arising from the mesenchymal cells. More than 50 different categories are reported, as stated by the World Health Organization.

STSs are rare tumors and represent about 1% of all cancers.^1^ Despite their low incidence, these malignancies are worrisome; in fact, about 25% of STSs develop distant metastases, representing the main factor leading to death, with a metastatic percentage that can reach about 50% for high-grade STSs.^2–4^ The main site of metastatization is the lungs, which account for about 80% of lesions.^5^

Prognosis of patients who develop metastases is generally: 3-year survival rate is lower than 50% for those undergoing surgical metastasectomy and lower than 20% in those who are not candidate to surgery. Thus, an imaging method, which potentially enables the prediction of metastases occurrence in this set of patients might be of high benefit. The median survival time after distant metastasis diagnosis is approximately 11.6 months.^2^ Identifying patients who are at a high risk of developing distant metastasis at an early stage could potentially allow implementing more effective therapies.^6^^,^^7^

Analysis of tumor heterogeneity on pathological samples obtained from biopsies may be challenging and the information may depend on which part of the tumor is sampled.^8^ Attempts to solve this issue and to obtain better information from STSs have been done with the so-called “Radiomics”, a field of imaging research which implies the analysis and extraction of large amount of data from medical images, using advanced quantitative characteristics and specific image-processing algorithms.^9^^,^^10^ As a matter of fact, with radiomics will be possible to avoid the biopsy and to identify the patients with greater risk of metastases or local recidive. In addition, the help of radiomics during the follow-up can support clinicians to predict the behavior of tumors.

Promising results have been recently reported in the use of radiomics in musculoskeletal oncology, being mainly aimed for investigating conventional statistical methods/machine learning algorithm for musculoskeletal sarcomas,^11^ for discriminating benign from malignant spine tumors,^12^ for predicting patient’s prognosis and treatment response.^7^^,^^13–15^ Nevertheless, a common problem of radiomics studies is related to the considerable number of imaging features that are evaluated by the software^9^ or to the poor understandability of the radiomic features in the clinical context. The first problem can have negative consequences on the accuracy of prediction models because some of these features may be redundant, useless, or highly correlated among each other.

Nowadays, various fields, including scientific research, data analysis, and medical diagnosis, utilize artificial intelligence (AI). Machine learning (ML)^16^ represents the most commonly exploited AI technique in the medical field as it enables the analysis of data, detection of patterns, and derivation of conclusions also without explicit input. However, this technique often encounters problems related to the complexity of AI, which are frequently used as “black boxes” due to the inability to comprehend the processing from input to output.^17^

For these reasons, the current study applies Formal Methods in the radiomics pipeline: these are based on mathematical logic and reasoning^18^^,^^19^ that allow to model the radiomic data of a patient and to verify if this satisfies the properties belonging to a disease state. Formal Methods are a group of logical and mathematical procedures used to confirm and demonstrate the accuracy and the correctness of a computer system or software application.

Compared to AI techniques, Formal Methods permit to:

- have a reduced dataset of patients and/or images for computing the model;
- produce an explainable and understandable model;
- easily reply the process, because a small number of parameters is required for the entire pipeline.

## OBJECTIVE

The purpose of this study was to provide a Formal Method to predict distant metastases and local recurrence in STSs of the extremities. The proposed technique was noninvasive and without the need for biopsy, indeed radiomic features were computed from nonenhanced magnetic resonance images (MRI). To the best of our knowledge, Formal Methods for STSs have never been analyzed in the literature.

## MATERIALS AND METHODS

### Dataset

An open-source deidentified database (http://doi.org/10.7937/K9/TCIA.2015.7GO2GSKS) was used as the source of our data.^7^^,^^20^ This set includes 51 histologically proven STSs of the extremities: all patients had pretreatment fluoro-D-glucose positron emission tomography (FDG-PET) and MRI scans between November 2004 and November 2011.

MRI protocols were not uniform among the patients. From the whole MRI dataset, we selected only MRI scans T2-weighted fat-saturated (T2FS) or short tau inversion recovery (STIR); patients were labelled “metastases/local recurrence” (group B) or “no metastases/no local recurrence” (group A) as clinical outcomes; 4 patients with upper limb soft tissue sarcoma were excluded. In terms of texture, T2FS and STIR images are considered to be similar, and therefore, they were grouped together under one category.^7^^,^^21^

Following these criteria, we included in our analysis a total of 47 patients from an overall number of 51 patients. Two different MRI exams (from 2 different patients) were used for modelling the property which will be automatically verified by a mathematical technique.

### Segmentation and feature analysis

Segmentations for exams were obtained from the above mentioned public database; for this study, segmentations included visible edema. Every single segmentation was visually valued by a radiologist with 7 years’ experience and modified if necessary. The 3D slicer software (4.13 version) was used for this step.^22^

All the radiomic features were computed using Pyradiomics 3.0.1 (https://pyradiomics.readthedocs.io), a library for radiomic features extraction from medical imaging,^23^ and a Python script developed by authors, which is compliant to the Image Biomarker Standardisation Initiative standard^24^ (IBSI).

The extracted radiomics features were of 7 main groups: (1) First Order (FOF) Features; (2) Gray Level Co-occurrence Matrix (GLCM) Features; (3) Gray Level Run Length Matrix (GLRLM) Features; (4) Gray Level Dependence Matrix (GLDM) Features; (5) Gray Level Size Zone Matrix (GLSZM) Features; (6) Neighbouring Gray Tone Difference Matrix (NGTDM) Features; (7) Shape Features 2 dimensional (2D). Definitions and detailed feature list are described in Pyradiomics feature documentation (https://pyradiomics.readthedocs.io).

The hyperparameter for feature extraction^25^ were as follows in Table 1; the remaining parameters were set to default. For each exam, features were extracted from each image separately.

**Table 1.** Description of the setting used for the feature extraction

Parameter	Option name	Value
Fixed bin count	binCount	50
Image normalization	normalize	True
Outliers to remove	removeOutliers	3
Interpolator for resampling	interpolator	sitk.sitkBSpline
Voxel size for resampling	resampledPixelSpacing	[0.6, 0.6, 0]
Forcing to texture calculation	force2D	True

**Figure 1.**
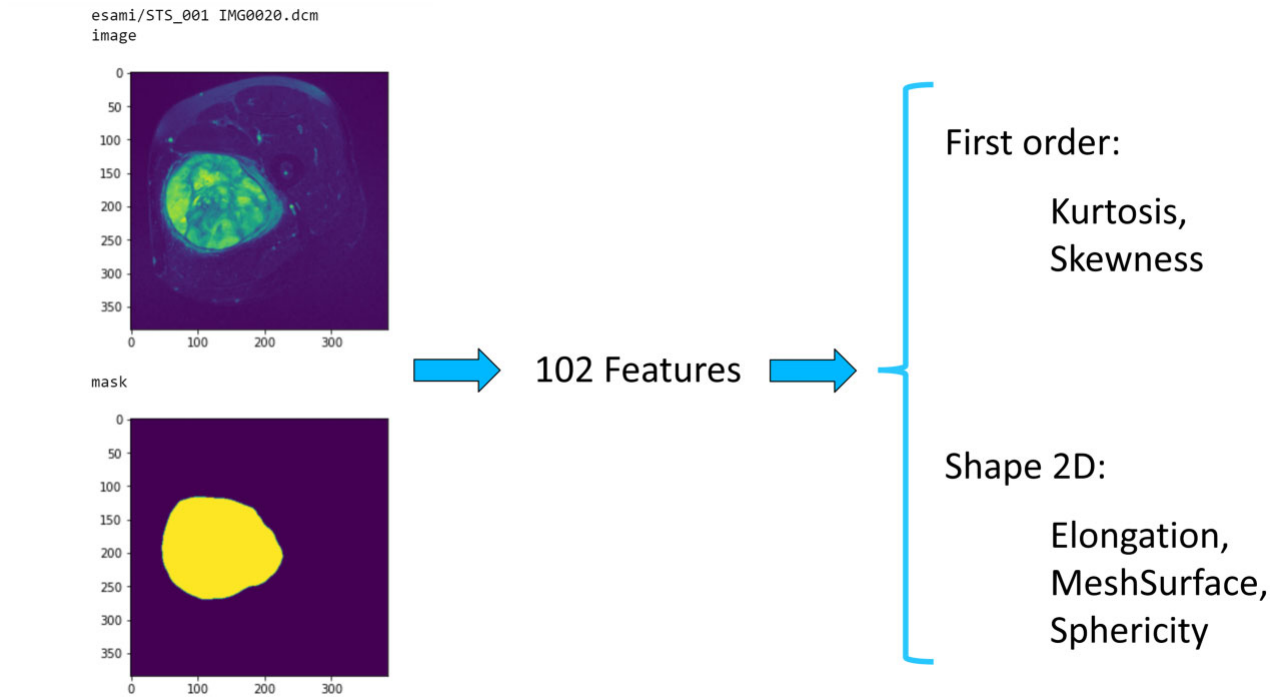
Radiomic pipeline: starting on the left, segmentation of a left tight pleomorphic sarcoma, feature extraction and 5 selected features.

Features used for Formal Method classifier were manually chosen, in order to describe the distribution of voxel intensities and shape characteristics, also with the support of Correlation Attribute Evaluation (Weka software version 3.8.5).^26^^,^^27^  Figure 1 shows an example of the Radiomic pipeline: 102 features were extracted from the segmentation of a left tight pleomorphic sarcoma, and finally were selected 2 first-order features and 3 Shape 2D features.

The next step was the discretization of extracted features, to simplify the translation in formal models. Authors divided the features in 3 different intervals: low, basal, and up with the equal-width partitioning. The discretized features were transformed into a formal model according to the Calculus of Communicating Systems (CCS), a process calculus introduced by Robin Milner.^28^^,^^29^ More detailed information about this process are reported in the following section.

### Formal Methods

Formal Methods^18^^,^^19^^,^^30^^,^^31^ are techniques derived from the Computer Science field to verify the correctness of critical informatic systems. Hence, thanks to the mathematical theory, this methodology allows to build formal and rigorous representation of a system. As a matter of fact, this methodology is widely used in Cybersecurity,^32–34^ Bioinformatics,^35^ and Computer Science to verify the safety of complex system behaviors where there is the possibility of economic losses or deaths (automated air traffic management or banking transitions).

**Figure 2.**
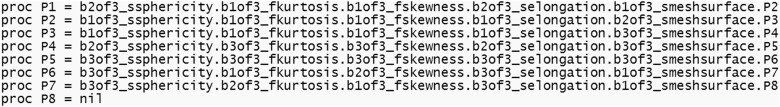
Example of formal model of a patient belonging to group A. It is the combination of the Sphericity, Kurtosis, Skewness, Elongation and Mesh Surface radiomic discretized features.

In the current study, instead of having a critical informatic system, authors considered the state of health of the patient: the methodology aims to verify the presence or the absence of a disease. After extracting the radiomic features, their numerical values were discretized in 3 levels; according to the keywords used in this methodology: a low level is represented by *b1of3*, a medium level is represented by *b2of3*, and the highest level is represented by *b3of3*. In order to create the formal model, radiomic features (Sphericity, Kurtosis, Skewness, Elongation, and Mesh Surface, as illustrated in the example below) were combined using the combination operator represented by the “.” symbol, as shown in Figure 2.

The entire sequence of clinical or radiomics features is represented by a “model” (one model is generated for each subject or patient). The pattern of a disease is represented through a “property” or “formula”; the property is computed by analyzing the common pattern of the disease with the aid of mathematical logic algorithms.

For instance, a pattern can be composed of 2 consecutive values *a* and *b*, followed, even if nonimmediately, by other 2 consecutive values *c* and *d*. Such a statement can conveniently be expressed in a temporal logic as:


prop F1=(min x=<a><b>F2 \/<−>X)prop F2=(min x=<c><d>tt \/<−>X).

**Figure 3.**
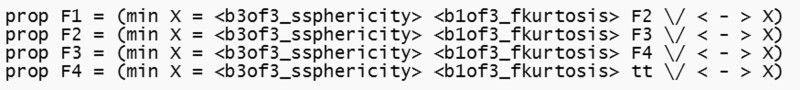
Example of formal property composed by the Sphericity and Kurtosis features.

The above representation is called “property” and, in case of a MRI exam, each row represents the discriminant radiomics feature values which correspond to the presence of a certain disease mark. In Figure 3, there is a practical example of property with a Shape feature called “Sphericity” and a First order feature called “Kurtosis”. Their numerical values are described through the discretization in 3 levels and different operators, which are:

- < and > are used for specify which action is performed;
- ∨ is used to perform an ‘OR’ combination of the values;
- *min X* is a function which define when the rule is satisfied;^36^
- *tt* or *ff* means the termination condition of the property.

**Figure 4.**
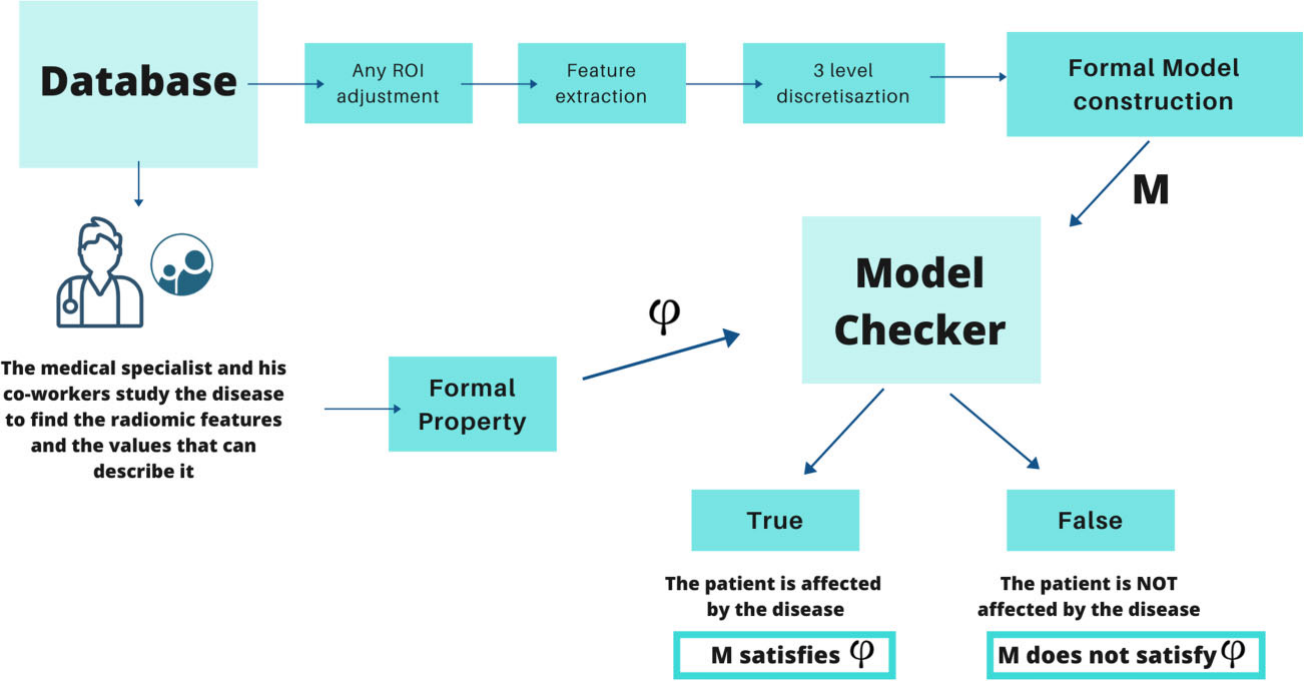
Explanation of the radiomic pipeline combined with the Formal Methods technique.

With the increase of radiomics studies on a certain disease, researchers can state (eg) that a low level of Kurtosis is indicative of a high risk of metastases or local recidive. Normally, the property is verified on a MRI exam independently of the number of slices contained. The agent responsible for verifying the absence or the presence of the property is called “Model checker”:^18^ it is a powerful automatic reasoning technique that allows a rigorous verification of the property on the model of the patient. Practically, this agent takes in input the model of the patient and the property of the disease status, verifying if the model satisfies the property; finally, the agent concludes with a simple result: the output is true if the model satisfies the property, otherwise it is false. If the output is true, this means the patient is affected by the disease status that the property describes (eg, the risk of metastases). If the result is false, researchers know the patient have a negligible risk of metastases and the methodology also return a counterexample with the explication why the model is considered false, which increments the understandability of the technique. In addition to the previous advantages, the use of Formal Methods can also help to localize the site of the sarcoma. Being a mathematical method, it allows to verify in which parts of the model the property is satisfied. In radiological terms, this functionality turns in a localization of the disease in the radiological exam. An example of the whole workflow, including radiomics and Formal Methods, is described in the Figure 4.

## RESULTS

### Clinical data

Our study population included 47 patients (23 men, 24 women; median age of 69 years, range 16–82 years). During the follow-up period, 21 patients did not develop metastases or local recurrence (group A), 26 patients developed metastases or local recurrence (group B); in particular, 23 patients developed metastases and 3 patients local recurrence.

The median time elapsed between the diagnosis and last follow-up was 790 days (range 458–2121 days) for group A, and the median time elapsed between the diagnosis and onset of metastases or local recurrence 216.5 days (range 66–1196 days) for group B. The median volume of the segmentations (including the visible edema) was 295.8 cm^3^ (range 28.8–2937.6 cm^3^) for group A, and 539.9 cm^3^ (range 109.3–3958.2 cm^3^) for group B. Regarding histological grade, 25 patients had high grade sarcoma (8 patients in group A—17 patients in group B), 15 patients had intermediate grade sarcoma (8 patients in group A—7 patients in group B) and 4 patients had low grade sarcoma (all these patients in group A); for 3 patients the histological grade was not available (1 patient in group A—2 patients in group B). Other relevant clinical parameters and treatment type are summarized in Table 2 and Table “DBInformation” provided in the Supplementary Materials section.

**Table 2.** Some clinical data for groups A and B

	Age [mean(min–max)]	Sex	Volume cm^3^ [median(min–max)]	No. of patients
Group A	48.5 (16–82)	13 F 8 M	295.8 (28.8–2937.611)	21
Group B	59.9 (34–78)	11 F 15 M	539.9 (109.3–3958.2)	26

MRI protocols were not uniform and only T2FS or STIR sequences were selected. Two exams were acquired in the sagittal plane, 4 exams were acquired in the coronal plane, and the remaining 41 exams were acquired in the axial plane. More details regarding to MRI acquisition protocols are reported in Table “MRIAcquisition” provided as Supplementary Materials.

In the public dataset, each individual patient did not have both STIR and T2FS sequences available; therefore, we selected the only available fluid-sensitive sequence.^7^^,^^21^

### Segmentation and feature analysis

After visually assessment, 43 segmentations were retained, and 4 segmentations were manually changed, in order to better delineate the profile of the lesion.

From segmentations, in total 102 radiomics features were extracted from T2FS or STIR images. For formal modelling, we considered only 5 features:^23^

- Kurtosis (First-Order feature): Kurtosis is a measure of the ‘peakedness’ of the distribution of values in the image region of interest (ROI);
- Skewness (First-Order feature): Skewness measures the asymmetry of the distribution of values about the mean value;
- Elongation (Shape feature 2D): Elongation shows the relationship between the 2 largest principal components in the ROI shape;
- Sphericity (Shape feature 2D): Sphericity measures the roundness of the shape of the tumor region relative to a circle;
- MeshSurface (Shape feature 2D): Mesh surface is derived from the approximated shape defined by the circumference mesh.

**Figure 5.**
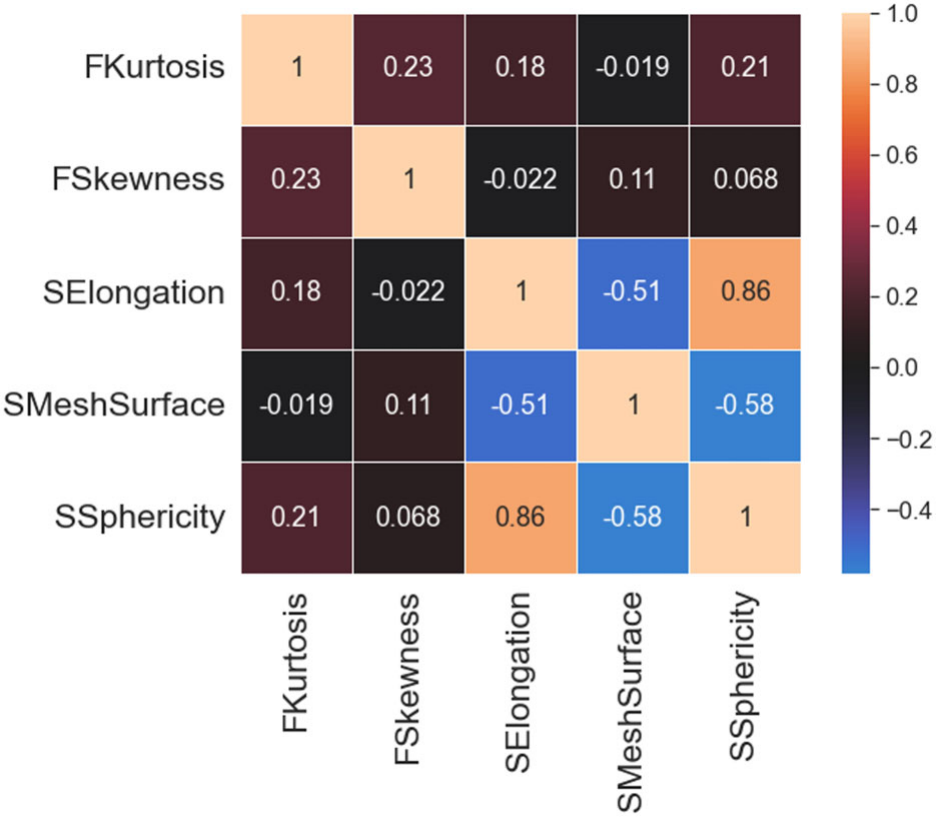
Intercorrelation among selected features.

Figure 5 shows intercorrelation among selected features, calculated according to Spearman correlation coefficient.

### Formal verification and statistical analysis

After generating the CCS models from all 47 patients, the disease property was generated by a radiologist and 2 computer science researchers, looking at 2 different exams, and it is shown in Table 3. Please note: in Formal Methods there is no division of training and testing, only the creation of the models, properties and their verification on the models. Formal Methods do not learn from any patterns or behaviors.

**Table 3.** Temporal logic properties used for the diagnosis of patients affected by sarcoma with local recurrence

*prop*	*F0 = F1 ∨ F10*
*prop*	*F1 = (min X = < b3of3sphericity> < b1of3kurtosis> F2 ∨ < - > X)*
*prop*	*F2 = (min X = < b3of3sphericity> < b1of3kurtosis> F3 ∨ < - > X)*
*prop*	*F3 = (min X = < b3of3sphericity> < b1of3kurtosis> F4 ∨ < - b3of3sphericity, b1of3kurtosis> X)*
*prop*	*F4 = (min X = < b3of3sphericity> < b1of3kurtosis> tt ∨ < - b3of3sphericity, b1of3kurtosis> X)*
*prop*	*F10 = (min X = < b3of3sphericity> < b1of3kurtosis, b2of3kurtosis> < b3of3meshsurface, b3of3elongation> F11 ∨ < - > X)*
*prop*	*F11 = (min X = < b3of3sphericity> < b1of3kurtosis, b2of3kurtosis> < b3of3meshsurface, b2of3meshsurface> F12 ∨ < - >X)*
*prop*	*F12 = (min X = < b3of3sphericity> < b1of3kurtosis, b2of3kurtosis> < b3of3meshsurface, b3of3elongation> F13 ∨ < - > X)*
*prop*	*F13 = (min X = < b3of3sphericity> < b1of3kurtosis, b2of3kurtosis>< b3of3elongation, b3of3meshsurface> F14 ∨ < -> X)*
*prop*	*F14 = (min X = < b2of3meshsurface, b2of3elongation> [-]ff ∨ < - > X)*

The following metrics were considered for evaluating the performance of the property to predict the development or not of metastases/local recurrence (group B vs group A): specificity, sensitivity (also called recall), accuracy, positive predictive value and negative predictive value. Intercorrelation among selected features was calculated with the Spearman correlation coefficient. Furthermore, the clinical utility indexes (CUI) were calculated to take into account both occurrence and discrimination.^37^ The value for CUI ranges from 0 to 1:

- excellent utility (CUI ≥ 0.81);
- good utility (CUI ≥ 0.64);
- satisfactory/fair utility (CUI ≥ 0.49);
- poor utility (CUI < 0.49);
- very poor utility (CUI ≤ 0.36).

This step of the analysis was performed with Python 3.7.11 version (Pandas 1.3.2 package).

Once the property was verified, the following metrics were computed:

- Sensitivity: 0.81;
- Specificity: 0.67;
- Accuracy: 0.74;
- Positive predictive value: 0.75;
- Negative predictive value: 0.74.

The positive CUI, calculated for the proposed formal method was 0.606; the negative CUI was 0.491: both these values have a satisfactory/fair utility value. Constructing the Confusion Matrix about the classification, we have:

- in the actual group A, consisting of 21 patients, 14 were correctly recognized, while 7 were classified as group B;
- in the actual group B, consisting of 26 patients, 21 of these were correctly classified while 5 were labeled as group A.

### Explainability

**Figure 6.**
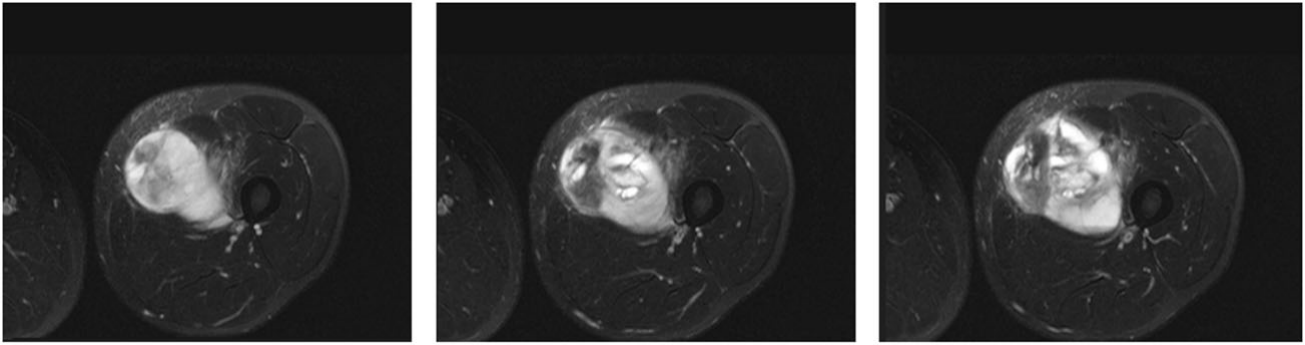
Three selected slices satisfying the property from a left tight sarcoma.

Combining Radiomics and Formal Methods allows to obtain a “second-virtual opinion” for radiologists and clinicians. In addition, this method can also localize the slices where the property is satisfied, giving a visual feedback, as shown in Figure 6. This can be very useful when facing difficult radiological exams where the main tumoral component is not clearly visible.

For example, in Figure 6 the localization method is used on patient STS_038 (from group B) and, in Table 4 are depicted which features values are aligned with the formula.

**Table 4.** Comparison between the values described in the property and those found in the radiological examination

	Sphericity	Kurtosis	Skewness	Elongation	Meshsurface
*Property*	High	Low	–	Medium/High	High
*Figure 6-1*	Medium	Medium	Medium	Low	High
*Figure 6-2*	High	Medium	High	Low	High
*Figure 6-3*	High	Low	Low	Medium	High

## DISCUSSION

This study provides a formal method to predict the development of distant metastases and local recurrence in STSs using MRI. The identified property obtained an accuracy of 0.74, a positive CUI of 0.606 and a negative CUI of 0.491. In the following section, we illustrate the choices in the materials and methods, the limitations, and recent articles regarding the same topic. For this work, 3 planes were considered: sagittal, coronal, and axial; axial plane was the most frequent acquisition plane. We decided to include all exams, even if with different planes, because current routine MRI protocols for STSs include all 3 orthogonal planes.

Regarding segmentations, it was preferred to include edema inside the segmentations, because STS cells are present histologically also in the tissues beyond the tumor.^38^ In addition, the ability to analyze tumor cells beyond the gross tumor volume has relevant implications such as in treatment.

Among all the features extracted through Pyradiomics,^39^ only 5 features were included in the property:

- Kurtosis (First-Order feature);
- Skewness (First-Order feature);
- Elongation (Shape feature 2D);
- Sphericity (Shape feature 2D);
- MeshSurface (Shape feature 2D).

As regards Kurtosis and Skewness, according to references 40–43, positive skewness and higher kurtosis can represent increased heterogeneity and poorer prognosis in several tumours; indeed, researchers in reference 44 showed that the presence of at least 2 out of 3 characteristics (heterogeneity, necrosis, and peritumoral enhancement) was a predictor of overall survival and metastasis-free survival in STSs. Regarding to Elongation and Sphericity, researchers in reference 45 included both features for their Radiomics-T2FS model, aimed to differentiate low-grade from high-grade STSs.

The principal limitations of the proposed study are as follows.

The first limitation is the relatively small sample size. However, differently from other AI techniques, the proposed approach does not require many exams because there is not a training step.

Regarding the second limitation, Elongation and Sphericity have a Spearman correlation coefficient of 0.86. It means redundant data can be present in the proposed property; anyway, we decided to retain them both on the basis of the above-mentioned article.^45^

The third limitation is the inclusion of patients with development of metastases (23 patients) and local recurrence (3 patients) in the same group B, considering these patients as a unique group.

The fourth limitation is due to differences in histology. According to references 46 and 47, the risk of distant metastasis in STSs ranges from 20% to almost 100% based on grading and histological type. Histology effect has not been investigated in this study and it could be explored in further studies.

The fifth limitation is the heterogeneity of MRI protocols and future works will verify the stability of the proposed approach through various image acquisition protocols.

Regarding the state-of-the-art, researchers in reference 48 trained a radiomics score for metastatic relapse-free survival in 35 patients with myxoid/round cell liposarcomas. The model, combining the radiomics score and relevant radiological features, achieved an AUC of 0.925.

Researchers in reference 49 found 5 contrast enhancement MRI radiomics models for predicting metastatic relapse-free survival, using 50 patients having high-grade sarcomas; the model with the highest integrative AUC obtained a value of 0.87.

Authors in reference 50 built a radiomics-based models to predict metastatic relapse at 2-years with a training data-set of 50 patients. On the testing cohort (20 patients), the best supervised model obtained an accuracy of 0.75.

In reference 51, the authors constructed and validated a radiomics method for prediction of distant metastasis in STSs. They used a training dataset with 54 sarcomas and a testing dataset with 23 sarcomas. The highest AUC and accuracy obtained were 0.902 and 0.913, respectively.

The above-mentioned articles^48^^,^^49^^,^^51^ considered only one histotype or the same MRI scanner. Differently, the proposed study included different histologies and different MRI scanners (further details regarding histological types and MRI scanner protocols are provided in “DBInformation” and “MRIAcquisition”—Supplementary Materials section).

Researchers in reference 7 used the same public data-set of the current study. They combined the texture features of FDG-PET and MRI imaging to assess lung metastasis risk in STSs on 51 patients; the model achieved an AUC, sensitivity and specificity of 0.984, 0.955, and 0.926, respectively.

Authors in reference 52 built a model for the prediction of lung metastases in STSs, by optimizing MR and PET image acquisition protocols. From the same public data-set of our study, the researchers selected 30 patients for their research and the model obtained an AUC of 0.89.

However, both the previous articles^7^^,^^52^ did not validate their results on independent datasets.

The above literature confirms the novelties of the proposed Formal Method approach, which can be considered as a valid alternative to other AI techniques. Moreover, even if the present results are slightly lower than the state of the art, the proposed method is highly-available, indeed it is based only on routine MRI protocols.

The aim of AI is to develop computer systems capable of reasoning and contributing to various fields, such as interpreting natural language,^53^ perceiving sensory information, and learning new information. These systems should emulate human intellect, performing tasks and improving their ability to perform them over time.

The most commonly used AI approaches are ML and deep learning (DL), which are often used interchangeably. ML^16^ is a broad category of approaches and algorithms that analyze data and draw conclusions. DL, on the other hand, is a subset of ML that uses artificial neural networks to analyze extremely complex data. Creating and utilizing a ML system can often pose challenges and difficulties. The first issue may be the lack of data, as ML requires a large amount of training data to be effective; but finding relevant and high-quality data to train the computer model can be difficult. Furthermore, overfitting and underfitting can arise due to noise or unrepresentative data in the training dataset, leading to ML models that are unable to accurately generalize predictions or classifications to new data. Even with a perfect dataset, interpreting the results of an ML system can sometimes be challenging because the model may be difficult to explain.^17^

Formal Methods can be utilized to analyze and evaluate the correctness and accuracy of computer systems by means of mathematical modeling of system behavior and the verification of specified properties. Formal Verification, Theorem Proving, Program Synthesis, and Model Checking are examples of Formal Methods techniques used to demonstrate a computer system's correctness with respect to specific requirements.^18^

By enabling errors in the software to be detected and resolved during development, formal approaches can enhance the dependability and security of computer systems, minimizing the probability of catastrophic errors or system failures.

As a conclusion, despite the limitations, the current study suggests that Formal Methods can provide beneficial assistance for personalized medicine. As a matter of fact:

- the availability of small datasets does not affect the robustness of the model and therefore the reliability of the results;
- the construction through mathematical and rigorous methods allows to understand the production and the meaning of the property (avoiding the risk of a “black box” approach);
- the entire process is supervised by radiologists and AI experts.

## CONCLUSIONS

The proposed approach, based on Formal Methods, can be an alternative tool to predict the risk of local recurrence and metastases in STSs. If the data are confirmed from further validation, this technique may assist physicians in choosing the appropriate treatment for STSs and potentially improve patient survival. Future works can be the development of these mathematical methods to extrapolate the objective characteristics of the disease independently of MRI scanners.

## Supplementary Material

ooad025_Supplementary_Data

## Data Availability

The data presented in this study are available on The Cancer Imaging Archive: http://doi.org/10.7937/K9/TCIA.2015.7GO2GSKS. The segmentations used in this study are available on request from the corresponding author.

## References

[1] Kransdorf MJ. Malignant soft-tissue tumors in a large referral population: distribution of diagnoses by age, sex, and location. AJR Am J Roentgenol 1995; 164 (1): 129–34. 7998525 10.2214/ajr.164.1.7998525

[2] Billingsley KG , Lewis JJ, Leung DH, Casper ES, Woodruff JM, Brennan MF. Multifactorial analysis of the survival of patients with distant metastasis arising from primary extremity sarcoma. Cancer 1999; 85 (2): 389–95. 10023707

[3] Brennan M. Soft tissue sarcoma: advances in understanding and management. Surgeon 2005; 3 (3): 216–23. 16076008 10.1016/s1479-666x(05)80044-7

[4] Stojadinovic A , Leung DH, Hoos A, Jaques DP, Lewis JJ, Brennan MF. Analysis of the prognostic significance of microscopic margins in 2,084 localized primary adult soft tissue sarcomas. Ann Surg 2002; 235 (3): 424–34. 11882765 10.1097/00000658-200203000-00015 PMC1422449

[5] Lewis JJ , Brennan MF. Soft tissue sarcomas. Curr Problems Surg 1996; 33 (10): 820–72. 8885853

[6] Komdeur R , Hoekstra HJ, van den Berg E, et al Metastasis in soft tissue sarcomas: prognostic criteria and treatment perspectives. Cancer Metastasis Rev 2002; 21 (2): 167–83. 12465756 10.1023/a:1020893200768

[7] Valliéres M , Freeman CR, Skamene SR, El Naqa I. A radiomics model from joint fdg-pet and MRI texture features for the prediction of lung metastases in soft-tissue sarcomas of the extremities. Phys Med Biol 2015; 60 (14): 5471–96. 26119045 10.1088/0031-9155/60/14/5471

[8] Longo DL. Tumor heterogeneity and personalized medicine. N Engl J Med 2012; 366 (10): 956–7. 22397658 10.1056/NEJMe1200656

[9] Kumar V , Gu Y, Basu S, et al Radiomics: the process and the challenges. Magn Resonance Imaging 2012; 30 (9): 1234–48. 10.1016/j.mri.2012.06.010 PMC3563280 22898692

[10] Lambin P , Rios-Velazquez E, Leijenaar R, et al Radiomics: extracting more information from medical images using advanced feature analysis. Eur J Cancer 2012; 48 (4): 441–6. 22257792 10.1016/j.ejca.2011.11.036 PMC4533986

[11] Gitto S , Cuocolo R, Albano D, et al Ct and mri radiomics of bone and soft-tissue sarcomas: a systematic review of reproducibility and validation strategies. Insights Imaging 2021; 12 (1): 1–14. 34076740 10.1186/s13244-021-01008-3 PMC8172744

[12] Gitto S , Bologna M, Corino VD, et al Diffusion weighted MRI radiomics of spine bone tumors: feature stability and machine learning-based classification performance. La Radiol Med 2022; 1–8. 10.1007/s11547-022-01468-7 PMC9098537 35320464

[13] Gnep K , Fargeas A, Gutiérrez-Carvajal RE, et al Haralick textural features on T2-weighted MRI are associated with biochemical recurrence following radiotherapy for peripheral zone prostate cancer. J Magn Reson Imaging 2017; 45 (1): 103–17. 27345946 10.1002/jmri.25335

[14] Bayanati H , E Thornhill R, Souza CA, et al Quantitative ct texture and shape analysis: can it differentiate benign and malignant mediastinal lymph nodes in patients with primary lung cancer? Eur Radiol 2015; 25 (2): 480–7. 25216770 10.1007/s00330-014-3420-6

[15] Fan M , Li H, Wang S, Zheng B, Zhang J, Li L. Radiomic analysis reveals DCE-MRI features for prediction of molecular subtypes of breast cancer. PLoS One 2017; 12 (2): e0171683. 28166261 10.1371/journal.pone.0171683 PMC5293281

[16] Mitchell TM , et al Machine Learning, vol. 1. New York: McGraw-Hill; 2007.

[17] Mézard M. Artificial Intelligence and Its Limits, vol. 49. France: EDP Sciences; 2018.

[18] Clarke EM Jr , Grumberg O, Kroening D, Peled D, Veith H. Model Checking. Cambridge, MA: MIT Press; 2018.

[19] Santone A. Heuristic search + local model checking in selective mu-calculus. IIEEE Trans Software Eng 2003; 29 (6): 510–23.

[20] Clark K , Vendt B, Smith K, et al The cancer imaging archive (TCIA): maintaining and operating a public information repository. J Digit Imaging 2013; 26 (6): 1045–57. 23884657 10.1007/s10278-013-9622-7 PMC3824915

[21] Zhao W , Huang X, Wang G, Guo J. PET/MR fusion texture analysis for the clinical outcome prediction in soft-tissue sarcoma. Cancer Imaging 2022; 22 (1): 7. 35022071 10.1186/s40644-021-00438-y PMC8756708

[22] Fedorov A , Beichel R, Kalpathy-Cramer J, et al 3d slicer as an image computing platform for the quantitative imaging network. Magn Resonance Imaging 2012; 30 (9): 1323–41. 10.1016/j.mri.2012.05.001 PMC3466397 22770690

[23] Van Griethuysen JJ , Fedorov A, Parmar C, et al Computational radiomics system to decode the radiographic phenotype. Cancer Res 2017; 77 (21): e104–7–e107. 29092951 10.1158/0008-5472.CAN-17-0339 PMC5672828

[24] Zwanenburg A , Valliéres M, Abdalah MA, et al The image biomarker standardization initiative: standardized quantitative radiomics for high-throughput image-based phenotyping. Radiology 2020; 295 (2): 328–38. 32154773 10.1148/radiol.2020191145 PMC7193906

[25] van Timmeren J , Cester D, Tanadini-Lang S, Alkadhi H, Baessler B, Radiomics in medical imaging—“how-to” guide and critical reflection. Insights Imaging 2020; 11 (1): 1. 32785796 10.1186/s13244-020-00887-2 PMC7423816

[26] Hall M , Frank E, Holmes G, Pfahringer B, Reutemann P, Witten IH. The weka data mining software: an update. SIGKDD Explor Newsl 2009; 11 (1): 10–8.

[27] Witten IH , Frank E, Hall MA, Pal C, Data M. Practical machine learning tools and techniques. In: Data Mining, vol 2. 2005: 4.

[28] Milner R. Communication and Concurrency, vol. 84. Englewood Cliffs: Prentice Hall; 1989.

[29] Brunese L , Mercaldo F, Reginelli A, Santone A. Formal methods for prostate cancer gleason score and treatment prediction using radiomic biomarkers. Magn Reson Imaging 2020; 66: 165–75. 31476359 10.1016/j.mri.2019.08.030

[30] Santone A , Vaglini G, Villani M. Incremental construction of systems: an efficient characterization of the lacking sub-system. Sci Comput Program 2013; 78 (9): 1346–67.

[31] De Francesco N , Lettieri G, Santone A, Vaglini G. Grease: a tool for efficient “nonequivalence” checking. ACM Trans Softw Eng Methodol 2014; 23 (3): 1–26.

[32] Voas J , Schaffer K. Insights on formal methods in cybersecurity. Computer 2016; 49 (5): 102–5. 10.1109/MC.2016.228 PMC5120363 27890940

[33] Poorhadi E , Troubitysna E, Dàn G. Formal modelling of the impact of cyber attacks on railway safety. In: Computer Safety, Reliability, and Security. SAFECOMP 2021 Workshops: DECSoS, MAPSOD, DepDevOps, USDAI, and WAISE, York, UK, September 7, 2021, Proceedings 40. Springer; 2021: 117–27.

[34] Cimitile A , Martinelli F, Mercaldo F, Nardone V, Santone A. Formal methods meet mobile code obfuscation identification of code reordering technique. In: *Infrastructure for Collaborative Enterprises (WETICE)*; 2017: 263–8.

[35] Ruvo GD , Nardone V, Santone A, Ceccarelli M, Cerulo L. Infer gene regulatory networks from time series data with probabilistic model checking. In: *IEEE/ACM 37th IEEE International Conference on Software Engineering*; Florence, Italy; 2015: 26–32.

[36] Emerson EA. Model checking and the mu-calculus. Descriptive Complexity and Finite Models 1996; 31: 185–214.

[37] Mitchell AJ. Sensitivity × ppv is a recognized test called the clinical utility index (CUI. +). Eur J Epidemiol 2011; 26 (3): 251–2. 21442261 10.1007/s10654-011-9561-x

[38] White LM , Wunder JS, Bell RS, et al Histologic assessment of peritumoral edema in soft tissue sarcoma. Int J Radiat Oncol Biol Phys 2005; 61 (5): 1439–45. 15817348 10.1016/j.ijrobp.2004.08.036

[39] Van Griethuysen JJM , Fedorov A, Parmar C , *et al*. Computational radiomics system to decode the radiographic phenotype. Cancer Res 2017; 77 (21): e104–7. 29092951 10.1158/0008-5472.CAN-17-0339 PMC5672828

[40] Davnall F , Yip CS, Ljungqvist G, et al Assessment of tumor heterogeneity: an emerging imaging tool for clinical practice? Insights Imaging 2012; 3 (6): 573–89. 23093486 10.1007/s13244-012-0196-6 PMC3505569

[41] Yang Z , Tang LH, Klimstra DS. Effect of tumor heterogeneity on the assessment of ki67 labeling index in well-differentiated neuroendocrine tumors metastatic to the liver: implications for prognostic stratification. Am J Surg Pathol 2011; 35 (6): 853–60. 21566513 10.1097/PAS.0b013e31821a0696

[42] Hockel M , Schlenger K, Aral B, Mitze M, Schaffer U, Vaupel P. Association between tumor hypoxia and malignant progression in advanced cancer of the uterine cervix. Cancer Res 1996; 56 (19): 4509–15. 8813149

[43] Castellano G , Bonilha L, Li L, Cendes F. Texture analysis of medical images. Clin Radiol 2004; 59 (12): 1061–9. 15556588 10.1016/j.crad.2004.07.008

[44] Crombé A , Marcellin P-J, Buy X, et al Soft tissue sarcomas: assessment of MRI features correlating with histologic grade and patient outcome. Radiology 2019; 291 (3): 710–21. 30964422 10.1148/radiol.2019181659

[45] Peeken JC , Spraker MB, Knebel C, et al Tumor grading of soft tissue sarcomas using mri-based radiomics. EBioMedicine 2019; 48: 332–40. 31522983 10.1016/j.ebiom.2019.08.059 PMC6838361

[46] Fletcher CD , Unni K, Mertens F. World Health Organization Classification of Tumours. Pathology and Genetics of Tumours of Soft Tissue and Bone. Lyon, France: IARC Press; 2002.

[47] Fletcher C. The evolving classification of soft tissue tumours: an update based on the new who classification. Histopathology 2006; 48 (1): 3–12. 16359532 10.1111/j.1365-2559.2005.02284.x

[48] Crombé A , Le Loarer F, Sitbon M, et al Can radiomics improve the prediction of metastatic relapse of myxoid/round cell liposarcomas? Eur Radiol 2020; 30 (5): 2413–24. 31953663 10.1007/s00330-019-06562-5

[49] Crombé A , Fadli D, Buy X, Italiano A, Saut O, Kind M. High-grade soft-tissue sarcomas: Can optimizing dynamic contrast-enhanced mri postprocessing improve prognostic radiomics models? J Magn Reson Imaging 2020; 52 (1): 282–97. 31922323 10.1002/jmri.27040

[50] Crombé A , Kind M, Fadli D, et al Intensity harmonization techniques influence radiomics features and radiomics-based predictions in sarcoma patients. Sci Rep 2020; 10 (1): 1–13. 32968131 10.1038/s41598-020-72535-0 PMC7511974

[51] Tian L , Zhang D, Bao S, et al Radiomics-based machine learning method for prediction of distant metastasis from soft-tissue sarcomas. Clin Radiol 2021; 76 (2): 158.e19–25. 10.1016/j.crad.2020.08.038 33293024

[52] Valliéres M , Laberge S, Diamant A, El Naqa I. Enhancement of multimodality texture-based prediction models via optimization of PET and MR image acquisition protocols: a proof of concept. Phys Med Biol 2017; 62 (22): 8536–65. 28872054 10.1088/1361-6560/aa8a49

[53] Chowdhary K. Natural language processing. In: Fundamentals of Artificial Intelligence. Midtown Manhattan, New York City: Springer; 2020; 603–49.

