# Correction to: Predicting risk of metastases and recurrence in soft-tissue sarcomas via Radiomics and Formal Methods

**DOI:** 10.1093/jamiaopen/ooad064

**Published:** 2023-07-28

This is a correction to: Roberto Casale and others, Predicting risk of metastases and recurrence in soft-tissue sarcomas via Radiomics and Formal Methods, *JAMIA Open*, Volume 6, Issue 2, July 2023, https://doi.org/10.1093/jamiaopen/ooad025

In the originally published version of this manuscript, affiliations were incomplete for the fourth, sixth and seventh authors. These should read: “Roberto Casale^1^, Giulia Varriano^2^, Antonella Santone^2^, Carmelo Messina^3,5^, Chiara Casale^4^, Salvatore Gitto^3,5^, Luca Maria Sconfienza^3,5^, Maria Antonietta Bali^1^, and Luca Brunese^2^” instead of: “Roberto Casale^1^, Giulia Varriano^2^, Antonella Santone^2^, Carmelo Messina^3^, Chiara Casale^4^, Salvatore Gitto^5^, Luca Maria Sconfienza^5^, Maria Antonietta Bali^1^, and Luca Brunese^2^”.

The emendations have been made in the article.

