# Supplementary material for: Predicting risk of metastases and recurrence in soft-tissue sarcomas via Radiomics and Formal Methods
Source: JAMIA Open. 2023 Apr 12;6(2):ooad025. doi: 10.1093/jamiaopen/ooad025 (PMC10097456; doi:10.1093/jamiaopen/ooad025)
Supplement: ooad025_Supplementary_Data [file ooad025_supplementary_data.zip › DBinformation.pdf]

Patient ID	Age	Sex	Histological type	MSKCC type	Site of primary STS	Grade	Volume cm^3	Treatment	Time – diagnosis to outcome (days)	Outcome (recurrence, mets)
STS_001	59	Male	pleiomorphic liposarcoma	Liposarcoma	left thigh	High	577,474	Radiotherapy + Surgery + Chemotherapy	266	Mets – arms
STS_002	61	Male	pleiomorphic leiomyosarcoma	Leiomyosarcoma	left buttock	High	58,6785	Radiotherapy + Surgery	--	--
STS_003	17	Female	epithelioid sarcoma	Other	right buttock	Intermediate	155,017	Radiotherapy + Surgery	--	--
STS_004	22	Female	malignant fibrous histiocytoma	MFH	right thigh	Low	295,8	Radiotherapy + Surgery	--	--
STS_006	64	Male	extraskeletal osteosarcoma	Extraskeletal bone sarcoma	right thigh	High	258,621	Radiotherapy + Surgery + Chemotherapy	198	Mets – lungs
STS_007	60	Female	spindle cell suggestive of myxofibrosarcoma	MFH	right buttock	Intermediate	537,701	Radiotherapy + Surgery	251	Mets – bones
STS_009	60	Male	myxofibrosarcoma - undifferentiated	MFH	right thigh	High	2372,08	Radiotherapy + Surgery + Chemotherapy	101	Mets – lungs
STS_010	60	Female	myxofibrosarcoma	MFH	left calf	High	373,287	Radiotherapy + Surgery	--	--
STS_011	56	Female	malignant solitary fibrous tumor	Other	right thigh	Intermediate	895,575	Radiotherapy + Surgery	--	--
STS_012	62	Female	sclerosing epithelioid fibrosarcoma	Fibrosarcoma	right thigh	Intermediate	109,341	Radiotherapy + Surgery	235	Mets – bones
STS_013	82	Male	dedifferentiated liposarcoma	Liposarcoma	left thigh	High	1536,49	Radiotherapy + Surgery	--	--
STS_014	56	Male	pleiomorphic leiomyosarcoma	Leiomyosarcoma	right thigh	Intermediate	534,22	Radiotherapy + Surgery + Chemotherapy	157	Mets – lungs
STS_015	34	Female	synovial sarcoma	Synovial sarcoma	right thigh	Intermediate	89,941	Radiotherapy + Surgery	--	--
STS_016	75	Female	spindle cell suggestive of pleomorphic liposarcoma	Liposarcoma	left thigh	Intermediate	924,637	Radiotherapy + Surgery	--	--
STS_017	54	Male	extraskeletal Ewing	Extraskeletal bone sarcoma	left thigh	High	728,127	Surgery + Chemotherapy	525	Mets – lungs
STS_018	68	Male	leiomyosarcoma	Leiomyosarcoma	left pelvis	Intermediate	112,631	Radiotherapy + Surgery + Chemotherapy	96	Mets – lungs
STS_020	78	Female	pleomorphic sarcoma	MFH	left thigh	High	3958,18	Radiotherapy + Surgery	66	Recurrence – regional
STS_021	49	Male	undifferentiated - pleiomorphic	MFH	right thigh	High	1040,02	Radiotherapy + Surgery + Chemotherapy	731	Mets – lungs
STS_022	65	Male	myxoid fibrosarcoma	MFH	left thigh	Intermediate	1348,27	Radiotherapy + Surgery + Chemotherapy	357	Mets – lungs
STS_023	65	Male	myxofibrosarcoma	MFH	left thigh	Intermediate	374,605	Radiotherapy + Surgery + Chemotherapy	98	Mets – lungs
STS_024	48	Male	pleiomorphic leiomyosarcoma	Leiomyosarcoma	left thigh	High	117,188	Radiotherapy + Surgery	--	--
STS_025	76	Male	malignant fibrous histiocytoma	MFH	right calf	High	28,8059	Radiotherapy + Surgery	--	--
STS_026	51	Female	liposarcoma	Liposarcoma	left thigh	High	377,03	Radiotherapy + Surgery + Chemotherapy	1196	Mets – spine
STS_027	62	Female	undifferentiated – high grade	MFH	left thigh	High	1025,44	Radiotherapy + Surgery + Chemotherapy	132	Mets – lungs
STS_028	70	Female	extraskeletal high grade osteogenic sarcoma	Extraskeletal bone sarcoma	left calf	High	204,32	Radiotherapy + Surgery	389	Mets – lungs
STS_029	47	Female	myxofibrosarcoma	MFH	left thigh	Intermediate	540,068	Radiotherapy + Surgery	--	--
STS_030	51	Male	liposarcoma, well diferentiated	Liposarcoma	right quadricep	Low	2937,58	Radiotherapy + Surgery	--	--
STS_031	70	Female	synovial sarcoma	Synovial sarcoma	right buttock	Intermediate	516,74	Surgery + Chemotherapy	94	Mets – lungs
STS_032	69	Male	pleiomorphic liposarcoma	Liposarcoma	right calf	High	158,037	Radiotherapy + Surgery	77	Mets – lungs
STS_033	74	Female	pleomorphic leiomyosarcoma	Leiomyosarcoma	left thigh	High	695,068	Radiotherapy + Surgery	167	Mets – lungs
STS_034	43	Male	poorly differentiated synovial sarcoma	Synovial sarcoma	right buttock	High	843,67	Surgery + Chemotherapy	76	Recurrence – local
STS_035	65	Female	undifferentiated – high grade	MFH	right thigh	High	31,01	Radiotherapy + Surgery	--	--
STS_036	24	Female	leiomyosarcoma	Leiomyosarcoma	right groin	High	597,544	Radiotherapy + Surgery + Chemotherapy	--	--
STS_038	63	Male	pleiomorphic liposarcoma	Liposarcoma	left thigh	High	542,014	Radiotherapy + Surgery	483	Mets – lungs
STS_039	44	Male	undifferentiated pleomorphic liposarcoma	MFH	left thigh	High	923,318	Radiotherapy + Surgery + Chemotherapy	189	Mets – lungs
STS_040	57	Female	epithelioid sarcoma with vascular differentiation	Other	right buttock	N/A	395,956	Radiotherapy + Surgery + Chemotherapy	73	Mets – lungs
STS_041	78	Female	leiomyosarcoma	Leiomyosarcoma	parascapusular	Intermediate	68,4405	Radiotherapy + Surgery	--	--
STS_042	16	Female	spindle cell suggestive of biphasic synovial sarcoma	Synovial sarcoma	left knee	Intermediate	36,2315	Radiotherapy + Surgery	--	--
STS_043	29	Male	mixoid liposarcoma	Liposarcoma	left thigh	Low	171,027	Radiotherapy + Surgery	--	--
STS_044	46	Male	mixoid liposarcoma	Liposarcoma	right thigh	Low	705,982	Radiotherapy + Surgery	--	--
STS_045	27	Male	extraskeletal osteosarcoma	Extraskeletal bone sarcoma	left adductur	High	501,407	Surgery + Chemotherapy	--	--
STS_046	57	Female	pleiomorphic undifferentiated sarcoma	MFH	right quadricep	High	866,321	Surgery + Chemotherapy	329	Mets – lungs
STS_047	62	Female	liposarcoma	Liposarcoma	right thigh	N/A	177,572	Radiotherapy + Surgery	--	--
STS_048	42	Female	pleiomorphic leiomyosarcoma	Leiomyosarcoma	left thigh	Intermediate	401,769	Radiotherapy + Surgery	--	--
STS_049	34	Male	liposarcoma - round cell	Liposarcoma	right calf	High	1087,05	Surgery + Chemotherapy	404	Mets – abdomen
STS_050	70	Female	pleiomorphic leiomyosarcoma	Leiomyosarcoma	left thigh	High	418,395	Radiotherapy + Surgery	418	Mets – lungs
STS_051	54	Male	synovial sarcoma	Synovial sarcoma	left poplietal fossa	N/A	173,567	Radiotherapy + Surgery	507	Recurrence – regional

NOTES

MSKCC type: As defined by an expert radiation-oncologist of sending institution using the classification of the Memorial Sloan-Kettering Cancer Center.
Time – diagnosis to outcome: Days elapsed between the date of diagnosis of primary STS (biospy) and the date of diagnosis of recurrence or metastases.
Status: No Evidence of Disease (NED), Alive With Disease (AWD), Dead (D).

N/A: Not Available
