# Supplementary material for: Predicting risk of metastases and recurrence in soft-tissue sarcomas via Radiomics and Formal Methods
Source: JAMIA Open. 2023 Apr 12;6(2):ooad025. doi: 10.1093/jamiaopen/ooad025 (PMC10097456; doi:10.1093/jamiaopen/ooad025)
Supplement: ooad025_Supplementary_Data [file ooad025_supplementary_data.zip › MRIAcquisition.pdf]

PatientName	Modality	Manufacturer	ManufacturerModelName	StudyDescription	SeriesDescription	PatientSex	SliceThickness (mm)	PixelSpacing - Adjacent row spacing (mm) adjacent column spacing
STS_001	MR	SIEMENS	Avanto	^THIGH	AXIAL SE T2 FAT SAT - RESEARCH	M	4	5,23E-01
STS_002	MR	Philips Medical Systems	Intera	L-SPINE	STIR longTE AX	M	6,364	5,95E-01
STS_003	MR	GE MEDICAL SYSTEMS	Signa HDxt	MRI PELVIS C- C	AX STIR	F	6	7,81E-01
STS_004	MR	Philips Medical Systems	Intera	RIGHT THIGH C+	T2ST RT - RESEARCH	F	5	1,01562
STS_006	MR	GE MEDICAL SYSTEMS	SIGNA EXCITE	MSK - Thigh (C+)	AX. T2 FSE FS - RESEARCH	M	5	1,64064
STS_007	MR	GE MEDICAL SYSTEMS	SIGNA EXCITE	MRI-PELVIS	Axial FSE/T2 Fatsat	F	6	7,81E-01
STS_009	MR	GE MEDICAL SYSTEMS	SIGNA EXCITE	MRI-RT THIGH	Axial FSE/T2 FS UPPER	M	5	8,59E-01
STS_010	MR	GE MEDICAL SYSTEMS	GENESIS_SIGNA	MRI LT LEG +C	3. AXIAL STIR - RESEARCH	F	6	5,86E-01
STS_011	MR	GE MEDICAL SYSTEMS	SIGNA EXCITE	MRI RT THIGH	sag FSE/T2 Fatsat	F	5	8,01E-01
STS_012	MR	GE MEDICAL SYSTEMS	GENESIS_SIGNA	MRI RT THIGH +C	5. AXIAL T2 F.S.	F	6	7,42E-01
STS_013	MR	Philips Medical Systems	Intera	LEFT KNEE/FEMUR	T2ST LT - RESEARCH	M	9	1,01562
STS_014	MR	GE MEDICAL SYSTEMS	SIGNA EXCITE	B MRI-RT THIGH	AXIAL FRFSE T2 Fatsat - RESEARCH	M	6	4,69E-01
STS_015	MR	GE MEDICAL SYSTEMS	Signa HDxt	IRM HANCHE DROITE C- C+	AX STIR	F	4	7,42E-01
STS_016	MR	SIEMENS	Avanto	IRM FEMUR/CUISSE GAUCHE C- C+	AX STIR iPAT - RESEARCH	F	10	1,171875
STS_017	MR	SIEMENS	MAGNETOM VISION	IRM FÉMUR/CUISSE GAUCHE C- C+ -MUSQ	HIP/T2_TURBO_STIR_RAPIDE_ - RESEARCH	M	8	7,81E-01
STS_018	MR	GE MEDICAL SYSTEMS	SIGNA EXCITE	MRI-PELVIS	AXIAL FRFSE T2 Fatsat Pelvis	M	5	7,81E-01
STS_020	MR	GE MEDICAL SYSTEMS	SIGNA EXCITE	B MRI THIGH C- LEFT	Coronal Fast Stir	F	6	8,98E-01
STS_021	MR	GE MEDICAL SYSTEMS	Signa HDxt	IRM EXTRU+00C9MITU+00C9S SANS & AVEC INFUSION	AX. STIR HAUT	M	6	1,1719
STS_022	MR	GE MEDICAL SYSTEMS	Signa HDxt	IRM FEMUR/CUISSE GAUCHE C+	AX STIR BILAT	M	8	8,40E-01
STS_023	MR	SIEMENS	Avanto	IRM extremite gauche sans et avec contraste	COR STIR	M	5	1,3671875
STS_024	MR	Philips Medical Systems	Intera	LEFT THIGH C+	T2ST LT	M	5	9,38E-01
STS_025	MR	SIEMENS	Sonata	ORTHO CHUS^OS LONGS	SAG STIR - RESEARCH	M	5	7,81E-01
STS_026	MR	Philips Medical Systems	Gyroscan Intera	LEFT THIGH C+	T2ST LT - RESEARCH	F	6	1,01562
STS_027	MR	GE MEDICAL SYSTEMS	SIGNA EXCITE	B LT THIGH	Coronal Fast Stir - RESEARCH	F	5	9,18E-01
STS_028	MR	GE MEDICAL SYSTEMS	SIGNA EXCITE	MRI-LT LEG	Axial FSE/T2 Fatsat - RESEARCH	F	6	6,45E-01
STS_029	MR	GE MEDICAL SYSTEMS	SIGNA EXCITE	MRI THIGH C+ LEFT	Coronal Fast Stir	F	5	8,98E-01
STS_030	MR	Varian Medical Systems	ARIA RadOnc	MRI-RT LEG	Axial T2 FS RT LOWER - RESEARCH	M	8	5,47E-01
STS_031	MR	GE MEDICAL SYSTEMS	SIGNA EXCITE	MRI-RT BUTTOCK	Axial T2 FS RT HIP/THIGH	F	5	5,47E-01
STD_032	MR	GE MEDICAL SYSTEMS	SIGNA EXCITE	B MRI-RT THIGH	Axial FSE/T2 FS ONLY TUMOR - RESEARCH	M	4	5,47E-01
STS_033	MR	GE MEDICAL SYSTEMS	SIGNA EXCITE	B MRI-LT THIGH	Axial FSE/T2 Fatsat - RESEARCH	F	6	8,20E-01
STS_034	MR	SIEMENS	Avanto	MSK^HIP	AXIAL STIR	M	8	1,3020834
STS_035	MR	SIEMENS	Espree	JAMBE^Reso-Concorde	AX STIR - RESEARCH	F	6	1,4648438
STS_036	MR	GE MEDICAL SYSTEMS	SIGNA EXCITE	B MRI-PELVIS	AXIAL FSE T2 - RESEARCH	F	5	7,81E-01
STS_038	MR	Philips Medical Systems	Intera	LEFT THIGH C+	T2SP LT - RESEARCH	M	6	1,01562
STS_039	MR	Philips Medical Systems	Intera	IRM - CUISSE GAUCHE	eSTIR_longTE SENSE W PICT-PLUS	M	5	5,99E-01
STS_040	MR	GE MEDICAL SYSTEMS	SIGNA EXCITE	IRM HANCHE DROITE C+	2-AX Stir irFSE H DRT - RESEARCH	F	6	4,30E-01
STS_041	MR	GE MEDICAL SYSTEMS	SIGNA EXCITE	MRI THIGH C+ RIGHT	Axial FSE/T2 Fatsat	F	5	4,69E-01
STS_042	MR	GE MEDICAL SYSTEMS	Signa HDxt	MRI EXTREMITIES WITHOU AND WITH CONTRAST - LEFT	AX IR - RESEARCH	F	7	3,91E-01
STS_043	MR	GE MEDICAL SYSTEMS	SIGNA EXCITE	B LT THIGH	Axial FSE/T2 Fatsat - RESEARCH	M	5	5,08E-01
STS_044	MR	GE MEDICAL SYSTEMS	SIGNA EXCITE	MSK - Thigh (C+)	AX. T2 FSE FS - RESEARCH	M	5	1,64062
STS_045	MR	GE MEDICAL SYSTEMS	GENESIS_SIGNA	e+1 HANCHE GAUCHE C+	3- O-AXI FAT T2 - RESEARCH	M	5	6,25E-01
STS_046	MR	GE MEDICAL SYSTEMS	SIGNA EXCITE	B RT THIGH	Axial FSE/T2 Fatsat - RESEARCH	F	6	8,59E-01
STS_047	MR	Philips Medical Systems	Gyroscan NT Intera	B KNEE RT	KNEE *AXT2SP	F		6,25E-01
STS_048	MR	GE MEDICAL SYSTEMS	SIGNA EXCITE	B MRI THIGH C+- LEFT	Axial FSE/T2 Fatsat	F	5	5,86E-01
STS_049	MR	GE MEDICAL SYSTEMS	SIGNA EXCITE	MRI-UNLISTED AREA	Axial FSE/T2 Fatsat LOWER TIB FIB - RESEARCH	M	7	4,30E-01
STS_050	MR	GE MEDICAL SYSTEMS	SIGNA EXCITE	IRM FEMUR GAUCHE C-/C+	AX STIR	F	4	3,91E-01
STS_051	MR	GE MEDICAL SYSTEMS	SIGNA EXCITE	B MRI-LT KNEE	AXIAL FSE T2 FATSAT - RESEARCH	M	5	7,03E-01

PixelSpacing - Adjacent column spacing (mm)	Rows (number of rows in the image)	Columns (number of columns in the image)	Scanning Sequence (SE: Spin Echo, IR: Inversion Recovery, RM: Research Mode)	SequenceVariant (SK: segmented k-space, SP: spoiled, MP: MAG prepared, OSP: oversampling phase, NONE: no sequence variant)	Patient Position (HFS: Head First-Supine, FFS: Feet First-Supine, FFP: Feet First-Prone)	ImageComments
5,23E-01	384	384	SE	['SK', 'SP', 'OSP']	HFS	LEFT
5,95E-01	336	336	IR	OSP	HFS	NaN
7,81E-01	512	512	IR	SK	FFS	NaN
1,01562	256	256	IR	OSP	FFS	NaN
1,64062	256	256	SE	NONE	FFS	NaN
7,81E-01	512	512	SE	SK	FFP	NaN
8,59E-01	512	512	SE	SK	FFS	NaN
5,86E-01	512	512	IR	SK	FFS	NaN
8,01E-01	512	512	SE	SK	FFS	NaN
7,42E-01	512	512	RM	NONE	FFS	NaN
1,01562	256	256	IR	OSP	FFS	NaN
4,69E-01	512	512	SE	['SK', 'OSP']	FFS	NaN
7,42E-01	512	512	IR	['SK', 'OSP']	FFS	NaN
1,171875	288	384	['SE', 'IR']	['SK', 'SP', 'MP', 'OSP']	FFS	NaN
7,81E-01	512	512	IR	OSP	FFS	LEFT FEMUR
7,81E-01	512	512	SE	SK	FFP	NaN
8,98E-01	512	512	IR	['SK', 'OSP']	FFS	NaN
1,1719	256	256	IR	SK	FFS	NaN
8,40E-01	512	512	IR	SK	FFS	NaN
1,3671875	256	256	['SE', 'IR']	['SK', 'SP', 'MP', 'OSP']	HFS	LEFT THIGH
9,38E-01	256	256	IR	OSP	FFS	NaN
7,81E-01	512	512	['IR', 'SE']	['SK', 'SP', 'MP', 'OSP']	FFS	RIGHT LEG
1,01562	256	256	IR	OSP	FFS	NaN
9,18E-01	512	512	IR	['SK', 'OSP']	FFS	NaN
6,45E-01	512	512	SE	SK	FFS	NaN
8,98E-01	512	512	IR	SK	FFS	NaN
5,47E-01	512	512	SE	['SK', 'OSP']	FFS	NaN
5,47E-01	512	512	SE	SK	FFS	NaN
5,47E-01	512	512	SE	NONE	FFS	NaN
8,20E-01	512	512	SE	SK	FFS	NaN
1,3020834	288	384	['IR', 'SE']	['SK', 'SP', 'MP', 'OSP']	HFS	RIGHT THIGH
1,4648438	192	256	['SE', 'IR']	['SK', 'SP', 'MP', 'OSP']	FFS	RIGHT LEG
7,81E-01	512	512	SE	NONE	FFS	NaN
1,01562	256	256	SE	OSP	FFS	NaN
5,99E-01	384	384	IR	SK	FFS	NaN
4,30E-01	512	512	IR	SK	FFS	NaN
4,69E-01	512	512	SE	SK	FFS	NaN
3,91E-01	512	512	IR	['SK', 'OSP']	FFS	NaN
5,08E-01	512	512	SE	SK	FFS	NaN
1,64064	256	256	SE	NONE	FFS	NaN
6,25E-01	512	512	SE	NONE	FFS	NaN
8,59E-01	512	512	SE	SK	FFS	NaN
6,25E-01	256	256	SE	OSP	FFS	NaN
5,86E-01	512	512	SE	SK	FFS	NaN
4,30E-01	512	512	SE	['SK', 'OSP']	FFP	NaN
3,91E-01	512	512	RM	NONE	FFS	NaN
7,03E-01	256	256	SE	OSP	FFS	NaN
